# Nucleotide sequence changes in human genome: detection by single-strand conformation polymorphism analysis

**Published:** 2004-02-01

**Authors:** Takao Sekiya

**Affiliations:** Mitsubishi Kagaku Institute of Life Sciences, 11 Minamiooya, Machida, Tokyo 194-8511

**Keywords:** Single-strand conformation polymorphisms (SSCP), point mutations, single nucleotide polymorphisms (SNPs), cancers, genetic diseases

## Abstract

Mobility shift of single-stranded DNA molecules with a single-base substitution in polyacrylamide gel electrophoresis due to a change of secondary and tertiary structures provides a simple, sensitive method, single-strand conformation polymorphism (SSCP) analysis, for detection of nucleotide sequence changes in DNA. The method with the quite unique principle can detect single-nucleotide substitutions, insertions or deletions of a short nucleotide sequence and loss of genes in human cancers and other genetic diseases. The great progress of the Human Genome Project has revealed thousands of genes associated with these diseases and led to an increasing need for detection of mutations and SNPs in large numbers of DNA samples. The recent development of high-throughput SSCP technologies will enable to meet this need even in a clinical setting.

## Introduction

Sequence of three billion base pairs in the human genome provides all the information necessary for human life. Characteristic differences between individuals are caused by tiny variations of the nucleotide sequence and therefore analyses of these polymorphisms make it possible to distinguish different persons. These polymorphic DNA changes include single-base substitutions (single nucleotide polymorphisms, SNPs) and different numbers of repeating units of a nucleotide sequence (variable number of tandem repeats, VNTRs).

Hereditary or sporadic changes of nucleotide sequences of the genome in patients of diseases also provide clues to distinguish abnormal cells from normal ones. In human cancers, for example, cumulative evidence indicates that accumulation of several genetic changes requires for the genesis of the diseases. The targets for the genetic changes in human cancers are oncogenes and tumor suppressor genes and these DNA alterations can be classified into two groups; one is alterations involving vast regions of DNA, such as amplifications, rearrangements and losses of genes, and the other is DNA changes involving short nucleotide sequences, such as single-base substitutions and deletions or insertions of one or a few nucleotides.

## Detection of genetic changes

Detection of these changes of nucleotide sequences is one of the most important issues of analyses of DNA. Vast DNA changes can be successfully detected by Southern blot hybridization.1) Gene amplifications can be detected by increases in the amounts of labeled probes hybridized to target DNA fragments, while gene rearrangements can be identified by the appearance of new restriction fragments. If two alleles in a particular individual can be distinguished by restriction fragment length polymorphism (RFLP) analysis, loss of heterozygosity (LOH) in cancer cells indicates deletion of the one of the two alleles of the gene. On the other hand, for detection of mutations in the second group, especially single-base substitutions, a special method other than Southern blot hybridization is required. Several techniques have been developed and used successfully, especially in conjunction with the polymerase chain reaction (PCR).

Determination of nucleotide sequences of genomic DNA is the most straightforward method for detection of single-base substitutions. Although an automated DNA sequencer based on capillary electrophoresis is quite high throughput and provides tremendous amounts of nucleotide sequence data, the most suitable use of nucleotide sequencing is still for identification of mutations detected by other methods.

A single nucleotide change can be detected by the ribonuclease (RNase) A mismatch cleavage method. The enzyme cleaves a labeled RNA probe at positions where it is mismatched to a target DNA or RNA sequence. The fragments produced and separated according to size can indicate the presence of a single-base substitution and the approximate position of the mismatched base pair.2)

In heteroduplexes between DNA probes and target DNA or RNA, mismatches at the point of mutations also make it possible to cleave the probes chemically. When DNA heteroduplexes are first modified with osmium tetroxide for T and C mismatches or with hydroxylamine for C mismatches and then incubated with piperidine, the probe DNAs at the modified bases can be cleaved.3) On the other hand, treatment of heteroduplexes with a water-soluble carbodiimide modifies unpaired G and T residues. When the duplexes thus modified are used as a template for primer extension with Taq DNA polymerase, termination of chain extension at the site of the carbodiimide-modified base results in generation of a fragment smaller than the full length product.4) By these chemical modification methods, single-base mutations were rapidly and successfully detected.

When a heteroduplex between mutant and wild-type DNA fragments is subjected to electrophoresis in a gel with an increasing gradient of denaturant (temperature, urea and/or formamide), a partly melted molecule that has very slow mobility in the gel is formed at a particular denaturant concentration being different among hetero- and homoduplexes. Formation of the partly melted molecules at different concentration of the denaturant results in different mobilities among DNA fragments.5) In this denaturing gradient gel electrophoresis (DGGE) method addition of a GC rich sequence of 40 base pairs (GC clamp) to one end of DNA fragments by the PCR can provide a relatively stable double-stranded region and help the formation of partly melted molecules improving the number of single-base changes that can be detected.6),7)

Hybridization of immobilized genomic DNA or PCR products with chemically synthesized oligonucleotide probes provides a simple method for identification of a single nucleotide change at a known position in the genome.8) These allele specific oligonucleotides (ASO) hybridize to perfectly matched unique sequences, but a single internal mismatch is sufficient to destabilize their hybridization to a target molecule.

All of the methods described above share the same principle based on the hybridization between complementary strands of DNA fragments. The method we have developed and described in this article is based on a completely different principle.

## Birth of single-strand conformation polymorphism (SSCP) method

In 1987, we had a chance to analyze DNAs from two independent tumors in the pancreas of a patient and could detect LOH at the *HRAS* locus in the DNAs from both tumors. To determine whether the remaining allele of the *HRAS1* gene contained a mutation, we cloned BamHI fragment carrying the gene from one of the pancreatic tumors. Nucleotide sequence analysis of the cloned DNA fragment revealed that the gene contained a silent base substitution at codon 27. Then we wanted to know whether the same allele remained in the other pancreatic tumor and decided to perform DGGE analysis using single-stranded DNA of *Pst*I fragment carrying exon 1 of the gene as a probe strand for formation of heteroduplexes. During this trial, electrophoresis in one of gels without denaturant provided signals with different mobility between ^32^P labeled *Pst*I fragments carrying the silent base substitution at codon 27 and that carrying the codon without the base substitution. This unexpected observation prompted us to hypothesize that the different mobility might be caused by a conformational change of single-stranded DNA due to a single-base substitution. Later, by determination of the total nucleotide sequence of the *Pst*I fragment, the mobility shift observed turned out to be due to different numbers of a repeating unit of 6 nucleotides, that was not know at that time, at the 5’-end of exon 1 of the *HRAS1* gene.9)

**Fig. 1:**
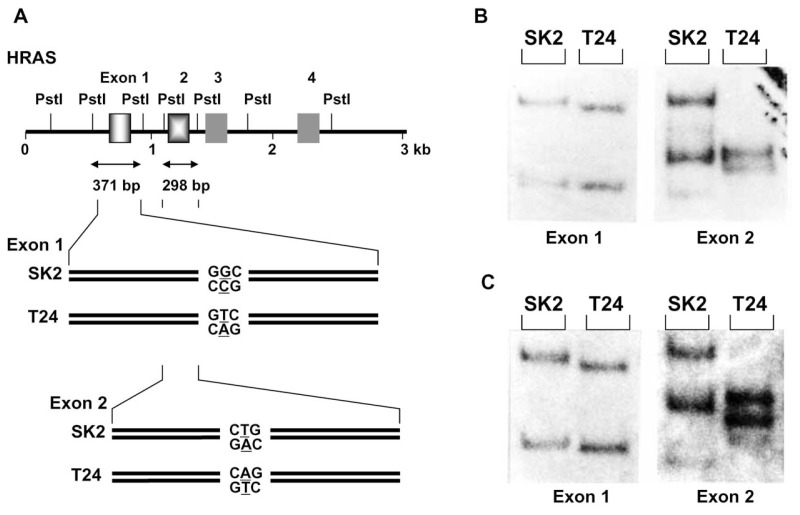
Mobility shift of single-stranded DNA fragments due to a single base substitution. (A) Fragments carrying exon 1 (371 bp) and exon 2 (298 bp) of the *HRAS1* gene from malignant melanoma SK2 cells and from bladder carcinoma T24 cells were obtained from plasmid clones (B) or genomic DNA (C) by digestion with restriction endonuclease *Pst*I. After denaturation, the fragments produced were subjected to electrophoresis in neutral polyacrylamide gel. Single-stranded DNAs were transferred to a nylon membrane and hybridized with the ^32^P-labeled DNA probe for exon 1 of the *HRAS1* gene (left panels in B and C) and the probe for exon 2 of the gene (right panels in B and C).

However, we could not discard the idea that single-stranded DNA fragments carrying the same nucleotide sequence but with a single-base substitution moved differently in non-denaturing polyacrylamide gel. Therefore, after denaturation to single-stranded DNA by heating in an alkaline solution, we subjected PstI fragments carrying exon 1 or exon 2 of the *HRAS1* gene of the cloned *Bam*HI DNA from human melanoma SK2 cells and that from human bladder cancer T24 cells to nondenaturing polyacrylamide gel electrophoresis. The former carries a mutated nucleotide sequence of CTG at codon 61 but a normal sequence of GGC at codon 12, while the latter has a mutated codon 12 of GTA but a normal sequence of CAG at codon 61 (Fig. 1A). The separated single-stranded DNAs in the gel were then transferred electrophoretically to a nylon membrane and hybridized with ^32^P-labeled DNA probes. The results clearly indicated that the pairs of separated strands of the *Pst*I fragment carrying exon 1 (Fig. 1B, left) and that of exon 2 (Fig. 1B, right) from the SK2 and T24 clones moved differently. Thus base substitutions could be successfully detected by mobility shift of single-stranded DNA from cloned DNAs. However, as an analytical method for detection of base substitutions it was necessary to confirm the same mobility shift of the target DNA fragments even in the presence of many other DNA pieces produced from genomic DNA. When digests of genomic DNA from SK2 and T24 cells by *Pst*I were similarly examined as the fragments from the cloned DNAs, exactly the same results were obtained (Fig. 1C).

Thus we found a new method for detecting single-base substitutions that based on mobility shift of single-stranded DNAs on gel electrophoresis by conformational changes due to single base substitutions. We named this feature of single-stranded DNA single-strand conformation polymorphism (SSCP).10)

## PCR-SSCP analysis

**Fig. 2:**
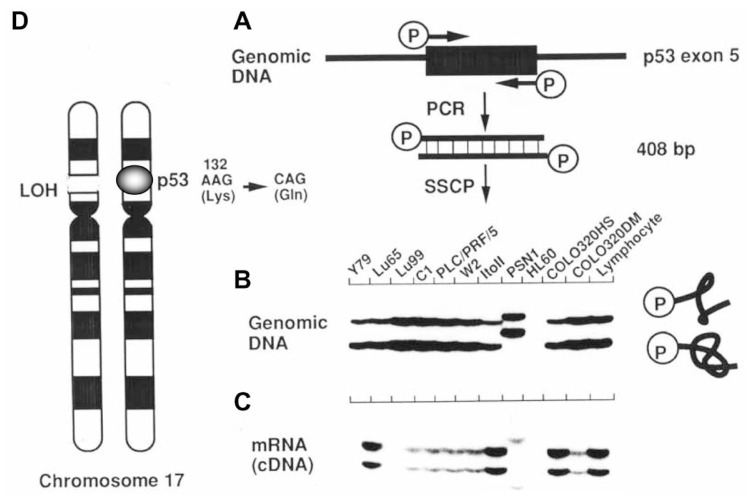
Analysis of DNA fragments carrying the nucleotide sequence of exon 5 of the *p53* gene. Genomic fragment of 408 bp was amplified and labeled by the PCR (A) and subjected to electrophoresis in 6% polyacrylamide non-denaturing gel without glycerol (B). The fragment was also amplified from the *p53* cDNA and analyzed by PCR-SSCP using the same gel with 10% glycerol (C). (D) Schematic presentation of the aberration of the *p53* gene observed in pancreatic cancer PSN1 cells.

Combined use of the polymerase chain reaction (PCR) and SSCP analysis have provided a simple and sensitive method for detection of point mutations.11)–13) In Fig. 2, PCR-SSCP analysis of a region containing exon 5 of the *p53* tumor suppressor gene in cancer cell lines is indicated. Using a set of two primers chemically synthesized and labeled with ^32^P at the 5’-end, the PCR was performed in the presence of genomic DNA and Taq DNA polymerase (Fig 2A). After thirty cycles of the reaction, a small portion of the reaction mixture was heated to denature double-stranded DNA products to single-stranded ones and then applied to 5% non-denaturing polyacrylamide gel with 10% glycerol. After electrophoresis the gel was dried on filter paper and exposed to X-ray film. On autoradiogram shown in Fig 2B, two signals corresponding to the separated complementary strands were observed and mobility shift of the strands was detected in a pancreatic cancer cell line PSN1. In this cell line, the signal only for the mutant allele was observed, indicating loss of the normal allele. In case of HL60 cell no signal was observed because of loss of both alleles of the *p53* gene.

When total RNA is isolated from cells and converted to cDNA with reverse transcriptase, PCR-SSCP analysis of cDNA thus prepared can detect the expression of the mutant *p53* gene (Fig. 2C).

A small portion of the dried gel corresponding to aberrant signals is cut out and soaked into water. A tiny amount of DNA eluted from the gel can be amplified by PCR using the same set of primers and then amplified DNA is subjected to nucleotide sequence analysis. These analyses revealed that in the PSN1 cell one of the two alleles of the *p53* gene was lost and in the remaining allele a single-base substitution at the position 132 in exon 5 resulted in alteration of Lys codon of AAG to Gln codon of CAG (Fig. 2D).

Thus PCR-SSCP analysis simultaneously reveals two typical changes of a tumor suppressor gene in cancer cells: loss of one allele and mutation in the remaining allele.

## Detection of DNA alterations by PCR-SSCP analysis

**Fig. 3:**
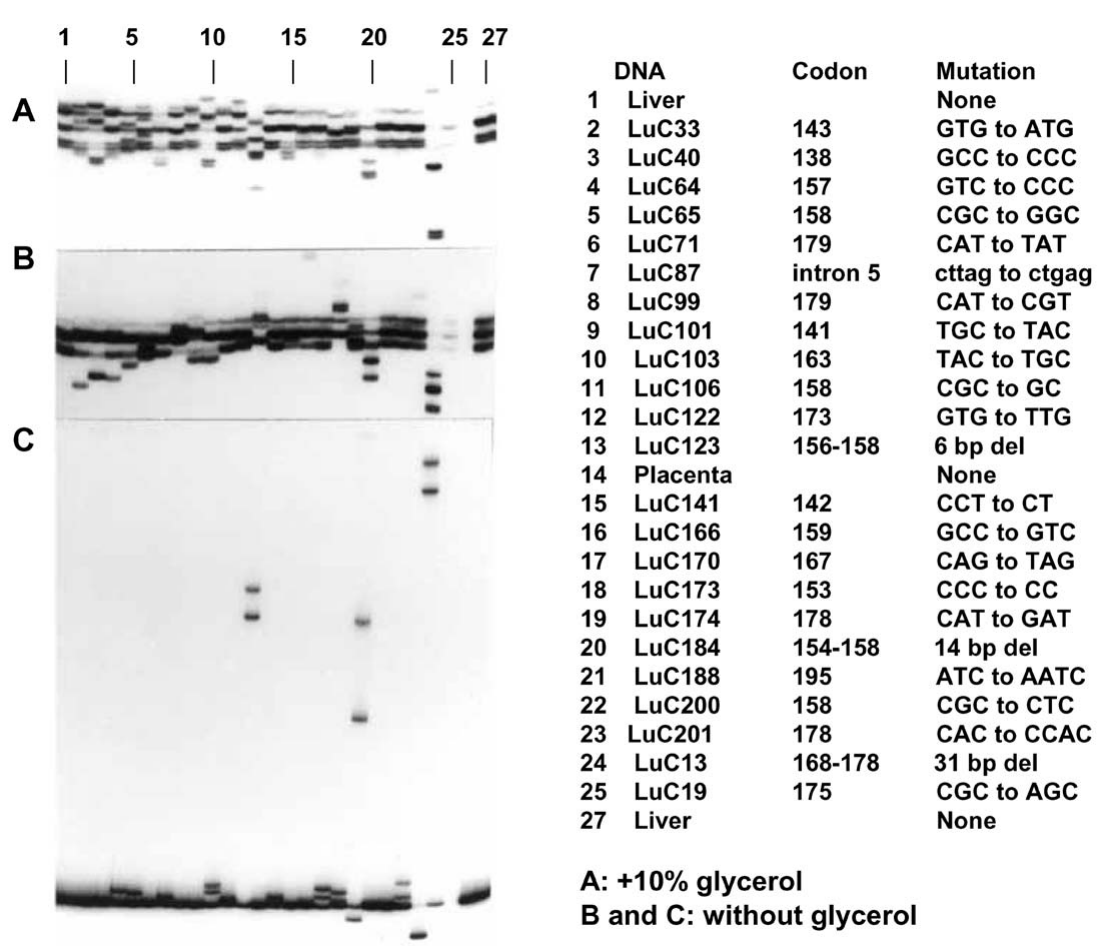
Detection of mutations of the *p53* genes in surgical specimens of non-small cell carcinoma of the lung by the PCR-SSCP method. (A) Mobility shifts observed in electrophoresis in 5% polyacrylamide gel with 10% glycerol. (B) Mobility shifts observed in electrophoresis in 5% polyacrylamide gel without glycerol. (C) Signals obtained in the lower part of the gel shown in B due to the formation of heteroduplexes and homoduplexes.

Results of the PCR-SSCP analysis of the same region of the *p53* gene shown in Fig. 2A in DNAs from surgical specimens of non-small cell lung cancers are shown in Fig. 3. Besides the signals observed in DNAs from normal tissues (Fig. 3A and B, lanes 1, 14 and 27), abnormal signals of mutant alleles were observed in cancer specimens. Because surgical specimens contain significant amounts of normal cells, signals from the mutant allele of the tumor cells are often weaker than those for the normal allele. The presence or absence of 5 or 10% glycerol in the gel gives significant difference in mobility of single-stranded DNAs and the presence of glycerol usually provides better resolution as shown in Fig. 3A. However, signals for some base substitutions are much clearer in the absence of glycerol (for example, lanes 4, 5 and 18 in Fig. 3B). Therefore, it is recommended to use both gels with and without glycerol.

In the lower part of the autoradiogram of the gel shown in Fig. 3B (Fig. 3C) signals observed are due to double-stranded molecules formed after denaturation of PCR products. Re-annealing of DNA fragments amplified from genomic DNA of heterozygous individuals results in a mixture of four duplexes, two homoduplexes and two heteroduplexes. Heteroduplexes have disordered structures with bubbles or bulges at the site of mismatched bases, and generally move more slowly in gel than homoduplexes.14) Heteroduplexes with a strand having deletion of nucleotides show very slow mobilities (Fig. 3C).

## Characteristics and advantages of PCR-SSCP analysis

The most important advantage of PCR-SSCP analysis is the detection of single-base substitutions at unknown positions of target DNA fragments. In our results, base substitutions at a position as close as 6 base pairs to the 3’-end of primers of 20 nucleotides in amplified DNA fragments of about 300-bp could be detected by mobility shift.

About the efficiency of detection of DNA alterations by PCR-SSCP analysis, with very few exceptions, all the single base-substitutions so far known could be detected and our results indicated that the efficiency of detection of single-base substitutions in fragments of less than 300 base pairs was more than 90% when both strands were labeled and were separated in gel containing glycerol. The mobility of a single-stranded DNA fragment is influenced by environmental conditions, such as the temperature of the gel and the presence or absence of glycerol in the gel. Therefore, it is important to optimize these conditions for better resolution.

Another great advantage of PCR-SSCP analysis is that different mobilities in polyacrylamide gel results in separation and purification of mutated fragments.15) As already described, after autoradiography of the dried gel, a tiny gel piece corresponding to the position of the separated band is cut out and the single-stranded DNA fragment is eluted. Then PCR of the eluted fragment provides a large amount of the fragment. By nucleotide sequence analysis of the PCR products, the target gene and the mutation can be identified.

Further the PCR-SSCP analysis can detect DNA abnormalities even in a very minor proportion of affected cells in a sample. Usually tumor tissues contain a significant amount of normal cells and in some surgical specimens tumor cells constitute less than 10% of the total cells. Point mutations in DNAs in such specimens cannot be detected by conventional methods. If the mobility shift observed is specific to cancer DNA, even a trace amount of signals indicates the presence of mutant alleles. A nucleotide substitution can be confirmed by direct sequencing of the DNA fragment eluted from the gel band with faint signals.

## Use of the PCR-SSCP method for genomic DNA analysis

We have successfully used PCR-SSCP analysis for detection of DNA alterations in human cancers. Our first application of the method was to analysis of *RAS* gene mutations in a large number of surgical specimens of lung cancers.12) We further used the SSCP method for analysis of the *RB* and *p53* tumor suppressor genes in a variety of human cancers.16)–19)

Soon after the method of SSCP was reported, it spread to other continents and played a critical role in identification of the causative genes of human hereditary diseases. The method was first used to detect and identify three different point mutations in the coding region of the cystic fibrosis transmembrane conductance regulator (*CFTR*) gene responsible for cystic fibrosis.20) Subsequently, it was used in identification of missense and nonsense point-mutations in the neurofibromatosis type 1 (*NF1*) gene,21) single-base substitutions resulting in creation of stop codons and small deletions in the familial adenomatous polyposis coli (*APC*) gene,22) a missense mutation in the fibrillin gene in patients with the Marfan syndrome23) and a single-base substitution in exon 2 of the HuP2 gene that encoded a member of the paired domain family of proteins and caused Waardenburg’s syndrome with hearing loss in a large Brazilian family.24) The PCR-SSCP method has been contributing to unveil a variety of disease causative genes up to now. For example, mutated *Ru2* and *Ru* genes in Hermansky-Pudlak syndrome,25) two mutations of the antimicrobial peptide hepcidin (*HAMP*) gene in severe juvenile hemochromatosis26) have been identified recently.

The PCR-SSCP analysis was further adapted for rapid diagnosis of mutations in a variety of human hereditary diseases, especially for analysis of the large size of genes, such as the ataxia-telangiectasia mutated (*ATM*) gene in the autosomal recessive disorder ataxiatelangiectasia. 27) Successful detection of mutations has been accomplished in disease genes such as the factor IX gene in severe hemophilia, the *β* -hexosaminidase *α*-subunit (*HEXA*) gene in Tay-Sachs disease, the aspartylglucosaminidase gene in aspartylglucosaminuria, the *β*- amyloid precursor protein gene in families multiply affected by Alzheimer’s disease, the phenylalanine hydroxylase (*PHA*) gene in phenylketonurea, the vitamin D receptor gene in vitamin D-dependent Rickets type II, the adult skeletal muscle sodium channel gene (*SCN4A*) in hyperkalemic periodic paralysis, the dystrophin gene in Duchenne muscular dystrophy, the catalase gene in acatalasemia patients, and many others. Recently, the PCR-SSCP method was successfully applied to find a new nitric oxide synthase gene (*NOS2*) associated with increased nitoric oxide production and protection from severe malaria in African children. 28) The method also unveiled a conformational change of mitochondrial Asn tRNA involved in a mitochondrial myopathy due to a mutation in the gene.29) Bacterium species infected in human patients was also identified by the method analyzing the 16S ribosomal RNA gene.30)

In cancer research, many new discoveries have been due to the use of the PCR-SSCP method for detection of critical or confirmatory point mutations. These include identification of a new tumor suppressor gene, the *APC* gene, apparently responsible for familial adenomatous polyposis coli,22) germline mutations in exon 9 or 8 of the Wilms’ tumor suppressor gene (*WT1*) in the Denys-Drash syndrome,31) a 17-base pair and one-base pair deletion in exons 4 and 6, respectively, of the *WT1* gene in hereditary Wilms’ tumor32) and identification of germline point-mutations in the promoter region of the *RB* gene.33) The *p53* gene in surgical specimens of human cancers has been analyzed extensively by the PCR-SSCP method and numerous mutations have been detected in all kinds of human cancers. Highly frequent *NRAS* gene mutation was detected in melanomas of patients with germ line mutations of the *CDNK2A* gene.34) *BRCA1* and *BRCA2* genes carrying many exons have been good targets to application of the PCR-SSCP method and revealed many disease-related mutations to understand hereditary and sporadic breast cancers.35)–37) The PCR-SSCP method could clarify inactivating *MSH6* mutations accounting for loss of mismatch repair in microsatellite instability in endometrial cancers,38) high prognostic value of *p16INK4* alterations in gastrointestinal stromal tumors39) and the relation of *cyclin D1* genotypes with biochemoprevention and progression rate to upper aerodigestive tract cancer.40)

Recently, the cDNA-SSCP analysis has been applied to assay of the expression of duplicated genes in plants.41)

## Nonisotopic SSCP

**Fig. 4:**
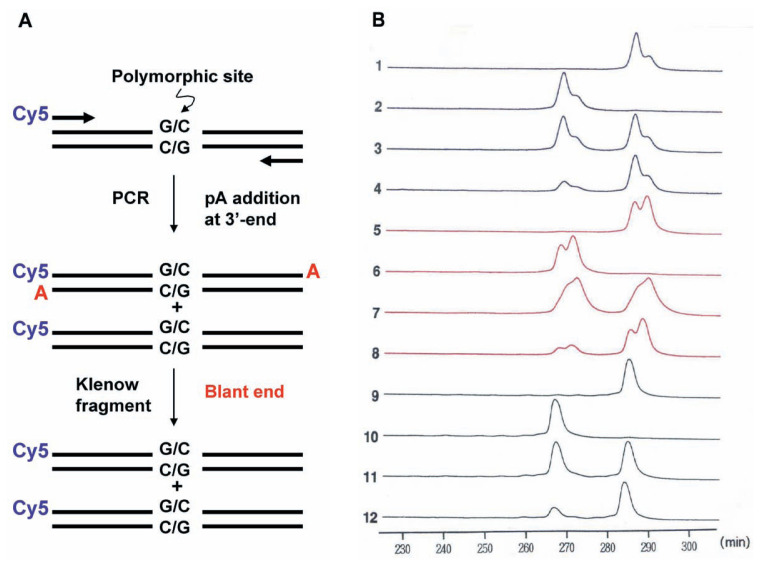
Fluorescent-based SSCP analysis of the PCR products amplified from the genomic DNAs of patients with colon carcinoma (A) by *Taq* DNA polymerase from two different suppliers (lanes 1–4, and 5–8 in B). Lanes 9–12 indicate blunt–end SSCP analysis of the PCR product shown in lanes 1–4 after blunting with the Klenow fragment. Lanes 1, 5 and 9 show DNA from homozygote carrying the *Hae*III-sensitive allele. Lanes 2, 6 and 10 show DNA from homozygote carrying the *Hae*III-resistant allele. Lanes 3, 7 and 11 show DNA from normal colonic mucosa of heterozygote. Lanes 4, 8 and 12 show DNA from colon carcinoma tissue from the heterozygote.

The extensive use of PCRSSCP analysis, especially in clinical laboratories, has been hampered by its requirement of radioactive nucleotides. To overcome this problem, methods for nonisotopic detection of SCCP by silver staining or ethidium bromide staining of single-stranded DNA in polyacrylamide gel was developed.42)–44) Fluorescence-labeled products of the PCR can be used successfully for SSCP analysis. The use of fluorescence-labeled primers for the PCR and analysis of SSCP with an automated DNA sequencer was as efficient as the conventional PCR-SSCP. 45) By this fluorescence labeling-based PCR-SSCP, a SNP of a G-C or C-G pair at a position in intron 1 of the *p53* gene was analyzed and applied to detection of loss of heterozygosity (Fig. 4). The target region was amplified by PCR using a fluorescence-labeled primer as one of a set of primers and four DNA samples from three individuals as templates, a homozygous one having the G-C allele, a homozygous one having the C-G allele, a heterozygous one having both G-C and C-G alleles and a tumor specimen of this heterozygous individual (Fig. 4A). During the PCR a portion of product molecules is known to be added by a pA residue at the 3’ end and give duplicated signals dependent on the DNA polymerase used as observed in Fig. 4B (lanes 1–8). When the PCR product was treated with Klenow fragment of *E. coli* DNA polymerase to remove the protruding A residue, each SSCP signal gave a single peak. The result shown in Fig. 4B lanes 9–12 clearly indicated that in the tumor of the heterozygous individual one of the alleles was lost.46)

## Recent advances in the PCR-SSCP technology

The tremendous progress of the Human Genome Project has been brought about by the transfer of DNA sequencing method from gel electrophoresis to capillary electrophoresis (CE). The transfer of gel-based technology to CE-based one has also provided a great advance of the PCR-SSCP method. The CE-SSCP method is quite high-throughput and is producing enormous amounts of data of hereditary and somatic mutations in human diseases and of base substitutions of SNPs in individuals.47)–49) CE instruments with up to 384 capillaries are commercially available and has been used for analysis of conformational changes of PCR amplified and most commonly fluorescence-labeled DNA using non-denaturing sieving matrices, such as commercial polymers of Genescan or native POP, linear polyacrylamides, short-chain polyacrylamide, short-chain dimethyl polyacrylamide and the co-polymer of short-chain poly(acrylamidedimethyacrylamide). For genome wide detection of nucleotide sequence changes, significant efforts at improving speed, accuracy and sensitivity of the SSCP method from the point of view of affecting parameters such as detection of signals, sample preparation, assay temperature, sieving matrices, buffer composition and pH and several others have optimized. For scanning of scattered unknown mutations for large genes such as *BRCA1* and *BRCA2* and/or screening of a large number of DNA samples for specific mutations, combined SSCP/heteroduplex analysis (HA) based on CE has been used.36) An automated tandem microchannel SSCP/HA capillary array electrophoresis is also the high-throughput, high-sensitivity mutation detection method.50)

## Conclusion

The great progress of the Human Genome Project has revealed thousands of genes associated with inherited disorders and cancers and led to an increasing need for detection of mutations and SNPs in large sample materials. The recent development of high-throughput SSCP technologies together with the quite unique principle of the SSCP method will enable to meet this need even in a clinical setting.

We believe that mobility shift of single-stranded DNA molecules with a single-base substitution is due to a change of secondary and tertiary structures. For high-throughput detection of nucleotide sequence alterations and understanding of the exact mechanism of SSCP, prediction of a particular conformation of a single-stranded DNA fragment from its nucleotide sequence is very important and helpful. From this point of view, the relation of CE-SSCP results and potential secondary structures predicted by an RNA/DNA-folding algorithm with DNA energy rules has been studied. The results give insight into the conditions of this measurement, especially temperature that should be optimized for best sensitivity of the SSCP method. The results also shed a new light on the understanding of the mechanism of SSCP.51),52)
